# Small Angle X-Ray and Neutron Scattering from Solutions of Biological Macromolecules.

**DOI:** 10.1107/S1399004714005719

**Published:** 2014-03-26

**Authors:** Leonard Michel Gabriel Chavas

**Affiliations:** a Center for Free Electron Laser science, DESY, Notkestrasse 85. Bldg 99, Hamburg, 22607, Germany

**Keywords:** book review, small-angle scattering, neutron scattering, biological macromolecule solutions

## Abstract

A review of *Small Angle X-Ray and Neutron Scattering from Solutions of Biological Macromolecules* by Dmitri I. Svergun, Michel H. J. Koch, Peter A. Timmins and Roland P. May.

*Small Angle X-ray and Neutron Scattering from Solutions of Biological Macromolecules* is a superb book that efficiently summarises both SAXS (small-angle X-ray scattering) and SANS (small-angle neutron scattering) techniques in a flow driven by the ambition to bring readers from an inexperienced background to a good practical knowledge and understanding of these scattering methods. Nobody else other than the experts in the field could write such an excellent work, the result of the authors’ several decades of experience in scattering experiments and implementations. Small-angle scattering is well established among specialists dedicated to the structural studies of macromolecules, and it is of no surprise that the IUCr Texts on Crystallography series welcomes such a textbook.

The topics of the book range from an introduction of the basics of small-angle scattering, through some specific details on the instrumentation behind SAXS and SANS experiments, data processing and analysis, to distinct examples of published real case studies. The text is divided into three parts, further subdivided in a total of nine chapters, covering the *Theory and Experiment* (Part I), the *Data Analysis Methods* (Part II), and the *Biological Applications of Solution Small Angle Scattering* (Part III). In the following, the content of the individual chapters will be reviewed.

The volume starts with an introductory chapter (*Introduction*, nine pages, 21 citations) that describes the general concept of small-angle scattering (SAS) and the role it plays in, and how it is appreciated by the structural biology community. It is then followed by Chapter 1 (*Basics of small angle scattering*, 14 pages, 19 citations), which gives a qualitative presentation of the SAS methods, only providing the readers with the information necessary for assimilating the fundamentals of optics. This short 14-page chapter sets the scene without ambiguities: the book is about scattering, and not diffraction. Chapter 1 pleasantly lacks the frequently found overflow of equations that often discourage young readers unfamiliar with the physics and mathematics behind the techniques; those equations will come in later chapters, once the entire context of the book is established.

Chapter 2 (*X-ray and neutron scattering instruments*, 38 pages, 63 citations) is devoted to the instrumentation behind SAXS and SANS experiments, giving the readers a practical aspect to the techniques. The choice by the authors of adding the experimental setup section right after the introduction and the basics rather than at the end of the book, as so often seen in other similar publications, builds up a clear overview on how SAS experiments should be approached. Starting with a description of the parameters required for these studies, the chapter includes an unusual description on how to switch between energy, wavelength and time of flight. A general understanding of X-ray and neutron sources is complemented by some examples of existing end-stations. The choice of the authors to end Chapter 2 with a comprehensive explanation of Spin Echo SANS (SESANS) implementations, describing how it could be coupled with SANS to cover a wider range of spatial resolutions, further identifies a method with considerable potential for development.

Targeting the experimenter, the various aspects of recording and analysing data are presented in Chapter 3 (*Experimental practice and data processing*, 25 pages, 26 citations). Here the reader can benefit from the expert advice from the authors who have contributed greatly to the spread of the methods, on the requirements of sample purity and a short-list of successful conditions prior to data collection. Some emphasis is given on the necessity to decipher properly all the properties of measuring instruments, pushing towards better data analysis for full background correction. General calibration aspects of the instruments are provided to conclude the practical view of the experiments.

Sensible readers can stop at Chapter 3, the rest of the textbook being all about the detailed equations that are at the heart of the technique, with clear examples of real case studies. Part II represents the reference for all those who need to understand and apply SAS, and is where this textbook carries its weight. Chapter 4 (*Monodisperse systems*, 59 pages, 170 citations) owes its length to the presence of countless equations (74), few figures, and an abundance of references. Somewhat disappointing is the last example in the chapter related to the label triangulation approach, which is lacking a deep explanation and mainly refers to a published work. It is not an isolated fact, however, and various examples within the book are too briefly described, referring to other sources and with incomplete descriptions, presumably but unrealistically leaving the reader to check a citation every few minutes.

While Chapter 4 is constructed around methods of extracting structural information from solutions containing particles of identical size, shape and mass, Chapter 5 (*Polydisperse and interacting systems*, 17 pages, 44 citations) defines polydisperse orders and more specific biological samples where solutions are not homogeneous and various multiple systems are present in a single solution. The overall situation is much more complex, and diverse difficulties arise, however, the description lacks detailed explanations on the most obvious approaches. The briefness of this chapter with respect to the relative length of the previous one on monodisperse systems contrasts with the relative importance of complex and polydisperse orders in biological systems.

Part III is dedicated to the presentation of practical approaches applied to the structural studies of biological systems in solution. This last section of the textbook starts at Chapter 6 (*Static structural studies*, 49 pages, 168 citations) by highlighting the strength of the technique when working on static samples. Within this chapter, the authors aim to provide the reader with a large set of examples to illustrate the wide variety of possible outputs, but inevitably there is a lack of detailed information and the reader is referred to further literature. The tendency to use SAS for more complicated systems is clearly emphasized, with a fair statement on the fact that SAS should by no means be used as the unique source of structural information, but rather it should be coupled to complementary techniques including macromolecular crystallography, electron microscopy, analytical ultracentrifugation, and others.

Chapter 7 (*Kinetic and perturbation studies*, 39 pages, 210 citations) presents a large overview of the applications related to time-resolved experiments. Similar to the previous chapter, this section is filled with references, which is a direct consequence of the growing interest shown by the structural biology community in this area of research. The applicability of the technique on light-induced systems at X-ray free-electron laser (XFEL) sources is also mentioned, but the applications of in-jet sample mixing are missing.

Chapter 8 (*Analysis of interparticle interactions*, 27 pages, 111 citations) starts with an introduction to the basic physical chemistry of interactions between particles, followed by further experimental data on protein–protein and nucleic acid interactions. For the reader who looks for inspiration rather than formal rigor, this section is of high interest as the study of the interactions of biological macromolecules remains a challenging area of research and remains open to further development.

The formal part of the textbook ends at Chapter 9 (*SAS in multidisciplinary studies*, 34 pages, 117 citation) by placing SAS in its global context. The authors attempt to balance SAS with other available techniques by highlighting their complementarity. The last section of the chapter emphasizes the need for the development of validation tools for the structures solved by SAS; indeed, to paraphrase the textbook, ‘given the inherent ambiguity of SAS data interpretation in terms of three-dimensional models, validation of these models becomes indispensable for making biologically relevant conclusions’.

The final chapter of the volume (*Conclusions and future prospects,* 2 pages) quickly reports on the limitations of the technique, the potential of XFEL sources for SAS studies, and the importance of automation in future SAS implementations. The book concludes with four appendices that describe the *Basic physics and mathematics of wave phenomena*, the *Spherical harmonics and their applications for SAS*, the *Interactions between spherical molecules*, and a list of *Web resources*, before ending with a brief index.

The scope of this textbook is strictly focused on SAS techniques for biological macromolecules, and covers all the aspects required to get an overview of the possibilities the techniques provide for structural and dynamic studies. Despite the clear, precise and rigorous presentation of the techniques all through the book, with instructive tables, simple and carefully chosen illustrations, and a very large variety of examples of real case studies, the reader is sometimes lost in a plethora of references, with a bibliography of nearly 1000 citations (some of which are cross-referenced) that requires additional effort. However, the bulk of the book remains of outstanding quality and clearly benefitted from the authors’ background in SAS and from their contribution to the field by many important publications.

The targeted audience of this textbook is the structural biologist willing to understand and apply the techniques to his or her research. This book is written by very experienced and knowledgeable experts in the field of small-angle scattering, and I warmly recommend it.

